# Structural Misalignment Between Regulatory Definitions of BCG-Unresponsive Non-Muscle-Invasive Bladder Cancer and Real-World Clinical Practice

**DOI:** 10.3390/healthcare14152303

**Published:** 2026-07-30

**Authors:** Philippe Pinton

**Affiliations:** Health & Life Sciences, Shiroito Co., Ltd., Tokyo 107-0061, Japan; philippe.pinton@shiroito.co.jp

**Keywords:** BCG-unresponsive disease, non-muscle-invasive bladder cancer, structural misalignment, operational criteria, health-system constraints, real-world evidence, guideline implementation, bladder-sparing therapies, classification frameworks, multilevel analysis, generalizability

## Abstract

**Highlights:**

**What are the main findings?**

- Identifies the structural and operational constraints that limit the applicability of current regulatory definitions of BCG-unresponsive non-muscle-invasive bladder cancer in real-world clinical practice.
- Demonstrates that the misalignment arises from system-level factors—maintenance uptake, surveillance variability, surgical access, and data granularity—rather than tumour biology or clinician behavior.
- Shows that a substantial proportion of patients cannot be classified under existing criteria because required exposure-based and time-dependent conditions are not met in routine practice.

**What are the implications of the main findings?**

- Highlights implications for therapeutic decision making, clinical trial eligibility, and interpretation of real-world evidence in structurally heterogeneous healthcare environments.
- Proposes context-specific operational criteria as a pathway to improve equitable access to bladder-sparing therapies and strengthen the validity of outcome research.

**Abstract:**

**Background:** Definitions of BCG-unresponsive non-muscle-invasive bladder cancer (NMIBC) have become central to therapeutic decision making and clinical trial eligibility. This perspective synthesizes regulatory frameworks with real-world observations to examine how structural conditions shape the applicability of current definitions. These definitions rely on structural prerequisites—adequate BCG exposure, routine maintenance therapy, standardized surveillance, timely access to early radical cystectomy, and complete tumour-level documentation—that are not consistently achievable across diverse health-care environments. This study examines the structural and operational factors that limit the applicability of current BCG-unresponsive criteria in real-world NMIBC care. **Methods:** A multilevel analysis was performed and integrated four complementary sources of evidence—regulatory frameworks, national claims datasets, multicenter clinical studies, and real-world practice observation—selected for their ability to capture distinct structural dimensions of NMIBC care. Operational assumptions embedded in contemporary definitions were compared with real-world treatment patterns. Structural barriers were categorized across macro-level system constraints, meso-level institutional practices, and micro-level clinical workflows. **Results:** As a result, a substantial proportion of patients cannot be classified under existing criteria because the exposure-based and time-dependent conditions required by regulatory definitions are not met in routine practice. Maintenance BCG is infrequently delivered, surveillance intervals vary widely, early radical cystectomy is limited by system-level and institutional factors, and key tumour-level variables required for classification are often missing in large-scale datasets. As a result, many patients cannot be reliably classified using existing criteria—not because of tumour biology or clinician behavior, but because the structural assumptions underlying the definitions are unmet. **Conclusions:** Current BCG-unresponsive criteria rely on structural conditions that are not universally present in real-world NMIBC care. These findings suggest that context-specific operational definitions, together with complementary strategies such as improving guideline implementation, enhancing data completeness, standardizing surveillance practices, and strengthening healthcare infrastructure, may help align regulatory expectations with real-world practice and support equitable access to bladder-sparing therapies.

## 1. Introduction

Bladder cancer represents a major global health burden, with more than 600,000 new cases annually according to WHO/IARC estimates [1], and its incidence is projected to rise substantially by 2040 [2]. Within this landscape, the definition of BCG-unresponsive non-muscle-invasive bladder cancer (NMIBC) has become a central determinant of contemporary management. It governs access to bladder-sparing therapies, eligibility for clinical trials, and the regulatory pathways through which new agents are approved.

This perspective integrates four complementary sources of evidence—regulatory frameworks, national claims datasets, multicenter clinical studies, and real-world practice observations—selected for their abilities to capture distinct structural dimensions of non-muscle-invasive bladder cancer care.

Contemporary definitions of BCG-unresponsive disease were developed within specific regulatory ecosystems, particularly through formal FDA guidance [3] and subsequently adopted by the EAU [4]. These frameworks assume a clinical environment in which maintenance BCG is routinely administered, surveillance is standardized, and early radical cystectomy is considered an acceptable and timely option. As observed by Claps [5], regulatory definitions developed within highly structured clinical ecosystems are difficult to operationalize in settings where the underlying structural assumptions—routine maintenance, standardized surveillance, and timely access to early cystectomy—are not consistently met. These operational assumptions represent structural conditions that vary substantially across healthcare systems.

In several real-world health-care environments, the assumptions are not reflected by actual practice. National guidelines may not incorporate the FDA/EAU definition [6], and real-world datasets often lack the tumor-level granularity required to operationalize exposure-based and time-dependent criteria [7,8]. Multicenter clinical data further reveal a striking paradox: high initial complete response rates coexist with low maintenance uptake and limited use of early cystectomy [9,10]. This combination exposes a systemic gap between regulatory expectations and real-world practice. While these observations are derived from Japanese datasets, similar patterns of heterogeneous surveillance, variable maintenance uptake, and incomplete documentation have been reported in other healthcare environments, suggesting that some aspects of this misalignment may be broadly relevant.

This challenge is consistent with broader observations from real-world datasets: claims-based sources such as MDV offer unmatched national coverage but lack the tumor-level granularity required to operationalize regulatory definitions [7,8], whereas EMR-based cohorts provide clinical depth but remain heterogeneous in documentation. These two ecosystems are therefore complementary, yet neither currently supports the strict application of FDA/EAU criteria.

This perspective proposes that the observed gap constitutes a clinical and regulatory grey zone, providing a conceptual framing for situations in which regulatory definitions cannot be operationalized due to structural constraints. By synthesizing regulatory frameworks, real-world datasets, and multicenter clinical observations, this study examines the structural challenges that limit the operational application of current BCG-unresponsive criteria in NMIBC care.

## 2. Guidelines and Real-World Data

Contemporary definitions of adequate BCG exposure and BCG-unresponsive disease originate from formal regulatory guidance [3] and are reiterated in major international guidelines [4]. These definitions presuppose a clinical environment in which patients receive adequate induction, structured maintenance, and uniform surveillance, and in which early radical cystectomy is considered a reasonable and timely intervention. These operational requirements depend on structural conditions—maintenance delivery, surveillance standardization, timely access to early radical cystectomy, and complete tumour-level documentation—that vary substantially across healthcare systems and directly shape the feasibility of applying regulatory definitions. They also assume the availability of granular clinical information—CIS status, tumour size, multiplicity, and confirmation of complete response—needed to apply temporal and exposure-based thresholds, as reflected in the EORTC risk models [11,12].

In several real-world health-care environments, these assumptions are not consistently met. National guidelines may not adopt the FDA/EAU definition [6], and maintenance therapy is administered infrequently [9,10]. This divergence was examined through a multilevel integration of regulatory frameworks, national claims datasets, multicenter clinical studies, and real-world practice observations. Surveillance intervals vary across institutions [6], and early cystectomy is often limited by cultural preferences, quality-of-life considerations, comorbidity, or institutional capacity [9]. As a result, the regulatory definition becomes difficult to apply, both clinically and conceptually. This difficulty reflects structural constraints, rather than deviations from guideline recommendations or clinician behavior, a pattern also observed in other healthcare environments where maintenance uptake, surveillance intensity, or documentation completeness vary. The gap between guideline expectations and real-world practice reflects a structural incompatibility between regulatory assumptions and the conditions present in real-world clinical environments.

Large-scale claims datasets illustrate this divergence. Sources such as MDV [7,8] offer unmatched national coverage, but lack the tumour-level granularity required to operationalize regulatory definitions. Key variables—including CIS status, tumour size, multiplicity, and documentation of complete response—are often absent from claims datasets, preventing the operational application of exposure-based and time-dependent criteria. EMR-based cohorts provide clinical depth, but remain heterogeneous in documentation, further complicating classification.

To examine the structural divergence between regulatory frameworks and real-world practice, we conducted a qualitative comparative synthesis of three guideline ecosystems: the FDA guidance, the EAU guidelines, and the JUA national guidelines. The FDA was selected as the primary regulatory reference because it provides the only formal definition of BCG-unresponsive NMIBC used in global drug development and clinical trial eligibility criteria. The EAU guidelines were included because they operationalize and disseminate this definition across European practice, representing an influential international clinical standard. The JUA guidelines were chosen as the national framework governing NMIBC care in Japan, where the real-world datasets and multicenter studies analyzed in this perspective were generated.

The EMA and PMDA were not included because neither agency provides an operational or exposure-based definition of BCG-unresponsive NMIBC. Unlike the FDA, which issued explicit guidance defining induction, maintenance, and time-dependent failure thresholds, the EMA and PMDA regulate drug approval pathways without establishing disease-level criteria for NMIBC. Their regulatory documents therefore do not offer a directly comparable framework for evaluating the structural assumptions—maintenance delivery, surveillance intensity, early cystectomy access, and tumour-level granularity—that underpin the FDA/EAU definition and that are central to this analysis. Including EMA or PMDA would not have added methodological value, as their documents do not address the operational conditions examined here.

Variables for comparison were selected a priori based on their relevance to operationalizing BCG-unresponsive criteria and their explicit presence in at least one of the guideline ecosystems. These included: (i) definitions of adequate BCG exposure (induction and maintenance), (ii) assumptions regarding maintenance delivery, (iii) surveillance schedules and intensity, (iv) recommendations for early radical cystectomy, and (v) requirements for tumour-level granularity (CIS status, size, multiplicity, and documentation of complete response). The synthesis was performed manually by the lead author, who extracted these variables from the most recent versions of the FDA guidance (2018), EAU NMIBC guidelines (2022), and JUA bladder cancer guidelines (2019), and aligned them with published multicenter Japanese datasets and claims-based sources.

Real-world data sources and studies were selected through a targeted literature search and expert knowledge of the Japanese NMIBC research landscape. The search focused on peer-reviewed multicenter studies and national-scale datasets reporting BCG treatment patterns, maintenance uptake, recurrence timing, cystectomy rates, and data granularity in Japan. Inclusion criteria were: (i) studies conducted in Japanese clinical settings, (ii) explicit reporting of BCG exposure and outcomes, (iii) use of either EMR-based or claims-based data structures, and (iv) relevance to at least one of the structural pillars examined in this perspective. Key studies by Miyake, Shibata, Tachibana, and Sasaki were identified through this process and complemented by the author’s prior work using MDV claims data. No formal systematic review was conducted; instead, the synthesis relied on a focused integration of high-relevance real-world evidence to illustrate how structural conditions constrain the operational application of regulatory definitions.

To contextualize the divergence between regulatory expectations and clinical reality, a comparative synthesis of these three guideline ecosystems is presented in Table 1.

Comparative alignment of international definitions of BCG-unresponsive NMIBC with real-world clinical practice. Divergences in maintenance BCG use [9,10], surveillance intensity [6], early cystectomy practice [9], and data granularity [7,8] illustrate the emergence of a grey zone in which international criteria cannot be operationalized reliably.

## 3. Real-World Data in Japan

Multicenter clinical datasets provide a detailed view of this divergence. Across several multicenter cohorts, 28% of patients met the FDA/EAU definition of adequate BCG exposure, and maintenance therapy was administered in 29% of cases, as reported in Miyake et al. and Shibata et al. [9,10]. These patterns reflect structural characteristics of the Japanese healthcare environment—maintenance delivery, surveillance intensity, surgical access, and documentation practices—although similar constraints have been reported in other settings with heterogeneous BCG uptake or variable data completeness. Despite high initial complete response rates, early failures are common and often occur before the temporal thresholds embedded in regulatory definitions [3,4]. Radical cystectomy is performed in only a small proportion of early failures, reflecting patient preference, comorbidity, and institutional practice patterns [9]. Tumour heterogeneity further complicates classification, particularly in patients with papillary tumors associated with CIS.

These findings illustrate that the clinical environment in which regulatory definitions were conceived—characterized by routine maintenance, standardized surveillance, and timely access to early radical cystectomy—differs structurally from real-world practice patterns observed in Japan. The patterns observed in multicenter datasets are not isolated. Although these observations derive from Japanese multicenter datasets, comparable divergences have been reported in other healthcare systems. Several independent studies have reported similar findings. Miyake et al. documented high induction completion, but low maintenance uptake and early recurrence patterns [9]. A separate analysis of induction therapy without maintenance using eight BCG instillations confirmed that real-world practice diverges structurally from guideline-recommended schedules [10,13]. Tachibana et al. further demonstrated that low-dose Tokyo-172 BCG regimens are widely used, adding another layer of heterogeneity [14]. Miyake et al. similarly reported low maintenance uptake, early recurrence dynamics, and limited early cystectomy across multiple centers, confirming that these patterns reflect national practice, rather than institutional variability [10].

Together, these studies confirm that the divergence between guideline expectations and real-world practice is structural rather than behavioral. This structural misalignment arises from exposure-based and time-dependent conditions embedded in regulatory definitions—adequate induction, maintenance delivery, standardized surveillance, and timely early cystectomy—that are not consistently achievable in routine practice. Taken together, these studies highlight a structural contradiction: current regulatory definitions rely on conditions that are not consistently present in real-world practice. This perspective proposes a conceptual framing of this inconsistency as a clinical and regulatory grey zone, in which definitions cannot be operationalized because their underlying structural assumptions are unmet. This perspective synthesizes existing evidence to clarify how structural conditions create a clinical and regulatory grey zone in which current definitions cannot be operationalized.

To illustrate the magnitude of this misalignment, Table 2 provides a qualitative comparison between regulatory thresholds and the published outcomes of a recent multicenter dataset [9]. The values reported in the table are extracted directly from peer-reviewed studies and do not derive from primary data analysis.

Quantitative comparison of regulatory requirements for BCG-unresponsive NMIBC with observed outcomes in multicenter real-world datasets. Divergences in adequate BCG exposure [9,10], maintenance use [9,10], recurrence timing [9], cystectomy rates [9], and data completeness [7,8] quantify the operational mismatch between regulatory criteria [3,4] and real-world practice.

Together, Table 1 and Table 2 illustrate the multidimensional gap between regulatory expectations and real-world NMIBC care. Regulatory definitions assume a high-compliance BCG environment with routine maintenance, standardized surveillance, and early cystectomy as an accepted option. In contrast, real-world practice is shaped by clinical norms, treatment patterns, and the characteristics of available data systems—including limited staging granularity, heterogeneous EMR documentation, and incomplete reporting of maintenance therapy—resulting in many patients being unable to meet the operational thresholds required for classification. This system-level misalignment creates a grey zone in which patients risk exclusion from therapeutic innovation not because of tumour biology, but because the prevailing definition was developed for clinical environments with different operational capacities, surveillance patterns, and treatment infrastructures.

## 4. Discussion

The integration of regulatory frameworks [3,4], macro-level claims data [7,8], micro-level clinical observations [9,10], and independent real-world studies [9,10,11,12,13,14] reveals a system-level misalignment between contemporary guideline definitions and real-world NMIBC practice. Regulatory definitions were developed within clinical ecosystems in which maintenance therapy is standard, surveillance is uniform, and early radical cystectomy is routinely considered. These conditions do not reflect real-world practice patterns observed in Japan, where maintenance uptake is low [9,10], surveillance intervals vary across institutions [6], and early cystectomy is infrequent due to quality-of-life considerations, comorbidity, and institutional capacity [9].

A central finding of this analysis is that the four structural conditions required to apply current definitions—adequate BCG exposure, maintenance therapy, timely early cystectomy, and tumour-level granularity—are inconsistently present in real-world Japanese practice, as documented in multicenter studies. Key variables needed to classify patients, including CIS status, tumour size, multiplicity, and documentation of complete response, are often missing in claims datasets [7,8], a limitation consistent with the observations of Miyake et al. [10]. Without tumour-level granularity—such as CIS status, tumour size, multiplicity, and documentation of complete response—international definitions cannot be applied statistically. When these variables are absent from claims datasets, patients cannot be assigned to any regulatory category, which effectively removes them from classification frameworks and from eligibility assessments based on those definitions.

## 5. The Four Structural Pillars of the Grey Zone in BCG-Unresponsive NMIBC

This section provides a conceptual synthesis of the four structural conditions embedded in contemporary definitions of BCG-unresponsive NMIBC. These conditions are derived from regulatory documents and published real-world studies, and are presented here to clarify why current definitions are difficult to operationalize in Japanese clinical practice.

1. Inadequate BCG Exposure in Real-World Practice

International definitions require adequate BCG exposure (complete induction plus maintenance). In multicenter datasets, only 28% of patients meet these thresholds, and maintenance therapy is administered in just 29% of cases [9,10]. More than two-thirds of patients are therefore excluded from the definition before any assessment of tumour response.

2. Early Recurrence Patterns Misaligned with Regulatory Timelines

Despite a high initial complete response rate (92.7%) [9], approximately half of recurrences occur before day 210 [9]. These early failures fall outside the temporal thresholds embedded in regulatory definitions [3,4]—thresholds that presuppose maintenance therapy rarely delivered in real-world practice—placing a substantial proportion of patients in a classification void.

3. Limited Use of Early Radical Cystectomy

Current definitions were designed to identify candidates for early radical cystectomy. In real-world datasets, only 17.6% of patients undergo cystectomy within 12 months of failure [9], reflecting cultural preferences, comorbidity profiles, and institutional practice patterns. This further disconnects real-world practice from the regulatory intent of the definition.

4. Absence of Tumour-Level Granularity in Claims-Based Data

Key variables required to apply regulatory criteria—CIS status, tumour size, multiplicity, and documentation of complete response—are frequently missing in claims datasets [7,8]. Miyake et al. [10] similarly demonstrated that tumour-level granularity is inconsistently captured in retrospective datasets, making statistical application of regulatory definitions unfeasible.

This pattern reflects a broader challenge in health-data infrastructure: real-world datasets not originally designed for oncology research often lack the tumour-level granularity required to apply exposure-based and time-dependent regulatory criteria, a limitation observed in Japan and in other settings relying on administrative data.

This conclusion derives from a structured review of published Japanese real-world datasets. Claims-based sources (e.g., MDV) provide national-scale coverage but lack tumour-level variables such as CIS status, size, multiplicity, and documentation of complete response [7,8]. EMR-based cohorts offer clinical detail, but remain limited by heterogeneous documentation and incomplete reporting of maintenance therapy [9,10]. Because each dataset captures only a subset of the variables required by exposure-based and time-dependent regulatory definitions, no single real-world data source currently contains the full information needed to operationalize these definitions.

Similar grey zones have been described in other clinical domains when guideline frameworks developed within idealized trial-based ecosystems are applied to heterogeneous real-world settings. Claps [5] noted that regulatory definitions developed within highly structured clinical ecosystems become difficult to apply when transposed into healthcare environments with divergent structural conditions. The structural challenges observed in NMIBC illustrate how regulatory definitions can lose operational meaning when the clinical and data conditions required for their application are not present in real-world practice.

The divergence across regulatory definitions illustrates that the operational boundaries of ‘BCG-unresponsive’ are evolving: the FDA maintains a narrow exposure-based definition, whereas the 2024–2025 EAU guidelines broaden the category to include incomplete responders and failures of combination regimens [3,4]. This reinforces the need for context-specific definitions in settings where the structural assumptions embedded in these frameworks are not met.

At the macro-data level, claims-based datasets lack the tumour-level granularity required to apply regulatory criteria [7,8]. At the micro-data level, clinical observations confirm low maintenance uptake, early recurrences, and limited early cystectomy [9,10]. These patterns arise from system-level constraints documented in Japanese real-world practice—such as limited tumour-level documentation, heterogeneous surveillance schedules, and institutional capacity—rather than from non-adherence to guideline recommendations. These findings demonstrate that the misalignment is a systemic incompatibility between regulatory assumptions and real-world practice.

This misalignment has direct therapeutic implications. Several bladder-sparing agents have been developed specifically for BCG-unresponsive disease—pembrolizumab [15], N-803 plus BCG [16], cretostimogene grenadenorepvec [17], and EG-70 [18]—yet patients may be excluded from these innovations not because of disease biology, but because the prevailing definition was developed for a different clinical ecosystem. This misalignment creates a risk of therapeutic inequity. When the operational definition of BCG-unresponsive disease cannot be applied because key variables are absent from real-world datasets, patients cannot be formally classified under regulatory criteria. As a result, individuals who exhibit the biological features of BCG failure may be excluded from therapies specifically developed for this condition, not because they are clinically ineligible, but because the definition cannot be operationalized in their clinical environment. This disconnect has direct implications for access pathways, trial eligibility, and the equitable diffusion of bladder-sparing innovations.

Understanding this structural misalignment is essential for interpreting real-world data, designing future studies, and ensuring equitable access to emerging bladder-sparing therapies. A context-specific approach to defining BCG failure—one that reflects local treatment patterns, cultural preferences, and data structures—is needed to bridge the gap between regulatory expectations and clinical reality.

## 6. Policy Implications

The findings of this study highlight the need for policy frameworks that recognize the structural constraints shaping NMIBC care. International definitions embed operational assumptions—routine maintenance BCG [9,10], standardized surveillance [6], and timely access to early radical cystectomy [9]—that are not consistently achievable across diverse health-care environments. This misalignment indicates the need for policy mechanisms that adapt these operational assumptions to the realities of Japanese practice, ensuring that eligibility criteria and access pathways do not exclude patients because structural conditions differ from those presumed in international guidelines. Applying these criteria without adaptation risks inequitable access to bladder-sparing therapies [15,16,17,18], inconsistent clinical decision making, and reduced validity of real-world evidence [7,8].

Policy action is required to develop context-specific operational criteria that reflect real-world practice patterns while maintaining alignment with international regulatory standards [3,4]. Such criteria would support more accurate patient classification by incorporating the variables that are consistently available in Japanese real-world datasets, rather than relying on tumour-level details that are structurally absent [15,16,17,18]. By aligning eligibility pathways with clinically observable patterns of BCG failure—such as recurrence timing and exposure history—these criteria would allow patients to be assessed using information that is reliably captured across institutions. Finally, using operational definitions grounded in real-world practice would enhance the interpretability of national outcome data by ensuring that classification rules are applied consistently across heterogeneous care environments. Strengthening system-level capacity—particularly in BCG supply stability, surveillance standardization, and surgical access—would further reduce structural barriers and improve the feasibility of guideline implementation.

These findings underscore the importance of integrating health-system realities into the design and adoption of clinical definitions, ensuring that regulatory frameworks remain applicable across diverse care environments.

Beyond operational alignment, reducing therapeutic inequities requires targeted policy measures that address the structural conditions limiting access to bladder-sparing innovations. Prioritizing the systematic capture of tumour-level variables within national data infrastructures would enable more patients to be accurately classified under regulatory definitions. Harmonizing surveillance schedules and maintenance documentation across institutions would reduce variability that currently restricts eligibility pathways. Finally, access frameworks for emerging therapies could incorporate clinically validated proxies for BCG failure when full definition criteria cannot be met due to data limitations. These measures would help ensure that patients are not excluded from innovations because of structural constraints unrelated to disease biology.

## 7. Conclusions

Across multiple layers of real-world evidence, a consistent pattern emerges: contemporary definitions of BCG-unresponsive NMIBC were conceived within clinical and regulatory ecosystems fundamentally different from those observed in Japanese real-world practice. The structural assumptions embedded in these definitions—adequate BCG exposure, routine maintenance therapy, standardized surveillance, and timely radical cystectomy—are not universally met, and available datasets do not capture the tumour-level granularity required to operationalize them.

This disconnect creates a clinical and regulatory grey zone in which established therapeutic decisions must be made without a framework aligned with local realities. Recognizing this misalignment is essential for interpreting real-world outcomes, designing future studies, and ensuring equitable access to emerging bladder-sparing therapies. Context-specific operational definitions, grounded in real-world treatment patterns and data structures, are needed to restore clinical relevance and support appropriate therapeutic decision making.

## Figures and Tables

**Table 1:** Comparative framework across FDA, EAU, and Japanese practice.

Dimension	FDA Definition (United States)	EAU Guidelines (Europe)	JUA Guidelines and Japanese Real-World Practice
Origin of the definition	Formal FDA Guidance (2018)	Adopts FDA definition almost verbatim	No formal “BCG-unresponsive” category in national guidelines
Adequate BCG requirement	≥5/6 induction + ≥2 maintenance instillations or second induction	Same thresholds as FDA	Induction common Maintenance rare (~29%)
Maintenance BCG	Assumed standard of care	Assumed standard of care	Low, heterogeneous, often omitted
Surveillance model	Structured, frequent cystoscopy	Structured, guideline-driven	Variable intervals, non-standardized
Early cystectomy	Expected in early failure	Recommended for high-risk disease	Underused and often declined for cultural and QoL reasons
Required tumor variables	CIS, size, multiplicity, histology, documented CR	Same granularity as FDA	Often missing in claims data (MDV) and inconsistently captured clinically
Applicability to claims data	Theoretically possible with granular data	Same limitations as FDA	Statistically inapplicable (missing CIS, size, multiplicity, CR)
Applicability in clinical practice	Designed for high-compliance BCG environments	Same assumptions as FDA	Clinically inapplicable in low-maintenance, low-cystectomy ecosystem
Impact on trial eligibility	Determines access to new agents	Same regulatory alignment	Majority fall “outside definition”

**Table 2:** Quantitative comparison of international criteria and Japanese data.

Parameter	FDA Requirement	EAU Requirement	JUA Guideline Position	JUA 2025 Dataset
Adequate BCG exposure	≥5/6 induction + maintenance	Same as FDA	Not formally defined	28% met FDA/EAU criteria
Maintenance BCG	Required	Required	Not standardized	29% received maintenance
Initial complete response	Required	Required	Recognized	92.7% CR after induction
Early recurrence threshold	≤6 months (HG) /≤12 months (CIS)	Same	No threshold	~50% before day 210
Early cystectomy	Expected	Recommended	Limited culturally	17.6% within 12 months
CIS documentation	Mandatory	Mandatory	Not systematic	Frequently missing
Tumor multiplicity/size	Mandatory	Mandatory	Not systematic	Not available in MDV
Eligibility for new therapies	Definition-based	Definition-based	No equivalent	Majority “outside definition”

## Data Availability

No new data were created or analyzed in this study.

## Abbreviations

AI	artificial intelligence
AUA	American Urological Association
BCG	Bacillus Calmette-Guérin
BCG-unresponsive	regulatory category defining failure after BCG according to FDA/EAU criteria
CIS	carcinoma in situ
CORE-001	trial of cretostimogene grenadenorepvec plus pembrolizumab
CR	complete response
EAU	European Association of Urology
EG-70	detalimogene voraplasmid
EORTC	European Organisation for Research and Treatment of Cancer
FDA	Food and Drug Administration
HG	high grade
JUA	Japanese Urological Association
KEYNOTE-057	pembrolizumab trial in BCG-unresponsive NMIBC
LEGEND	phase 2 trial of EG-70
MDV	Medical Data Vision
ML	machine learning
NMIBC	Non-muscle-invasive bladder cancer
QoL	quality of life
QUILT 3.032	trial of N-803 plus BCG
RC	radical cystectomy
RWD	real-world data
RWE	real-world evidence
TURBT	transurethral resection of bladder tumour
